# Case Report: A Malignant Liver and Thoracic Solitary Fibrous Tumor: A 10-Year Journey From the Brain to the Liver and the Spine

**DOI:** 10.3389/fsurg.2020.570582

**Published:** 2020-12-03

**Authors:** Min Mao, Lei Zhou, Chaojun Huang, Xudong Yan, Shuo Hu, Huabin Yin, Qinghua Zhao, Dianwen Song

**Affiliations:** Department of Orthopedics, Shanghai General Hospital, Shanghai Jiao Tong University School of Medicine, Shanghai, China

**Keywords:** solitary fibrous tumor, liver, spine, metastasis, malignant tumor

## Abstract

Solitary fibrous tumors are rare neoplasms that originate from mesenchymal tissues and have been found to occur in any site, including the spine and liver. Although most of solitary fibrous tumors have benign features, only 10–20% are malignant and prone to metastasis. No previous reports have described the malignant and metastatic Solitary fibrous tumor arising in both of the liver and thoracic vertebrae. In this article, we present the case of a 60-year-old woman who underwent gross total resection of a meningeal tumor in 2007. She presented 10 years later with a thoracic vertebral mass that caused relentless pain and a lesion in the right lobe of liver. She underwent marginal excision of the T3 tumor with T2-4 pedicular screw fixation in March 2017, then right hemi-hepatectomy was performed to remove the liver lesion in June 2017. Both of the lesions were confirmed to be a metastatic and malignant tumor after surgery. The literature lacks randomized controlled trials and large studies that define the natural history of malignant solitary fibrous tumors and recommendations of precise management plan for the disease. However, the best choice for treatment is gross total resection, which probably provide the optimal treatment to achieve long-term disease-free survival.

## Introduction

Solitary fibrous tumors (SFTs) are rare mesenchymal tumors derived from mesenchymal tissues and were first reported by Klemperer and Rabin in 1931 (1). Although SFTs mainly occur in the pleura, a few cases have been reported occurred in extra pleural organs, including the spine (2–4). According to World Health Organization classification of the central nervous system in 2016, SFTs and hemangiopericytomas were classified as the same tumor (5). However, to the best of our knowledge, SFTs located in the spine are relatively rare and were first reported by Carneiro et al. (6). SFTs can occur throughout the spine, and the thoracic segment (56.3%) represents the main lesion sites. Although most SFTs are benign and slow-growing, 10–20% of SFTs are malignant (7, 8). Unlike benign SFTs, malignant SFTs behave more aggressively, have a symptomatic presentation, are more likely to recur and metastasize and have a poor prognosis. Metastases to the spine from meningeal SFT are very rare. In this report, we report the rare case of meningeal SFT that metastasized to the thoracic spine and liver 10 years after the initial diagnosis.

## Case Report

**Figure 1:**
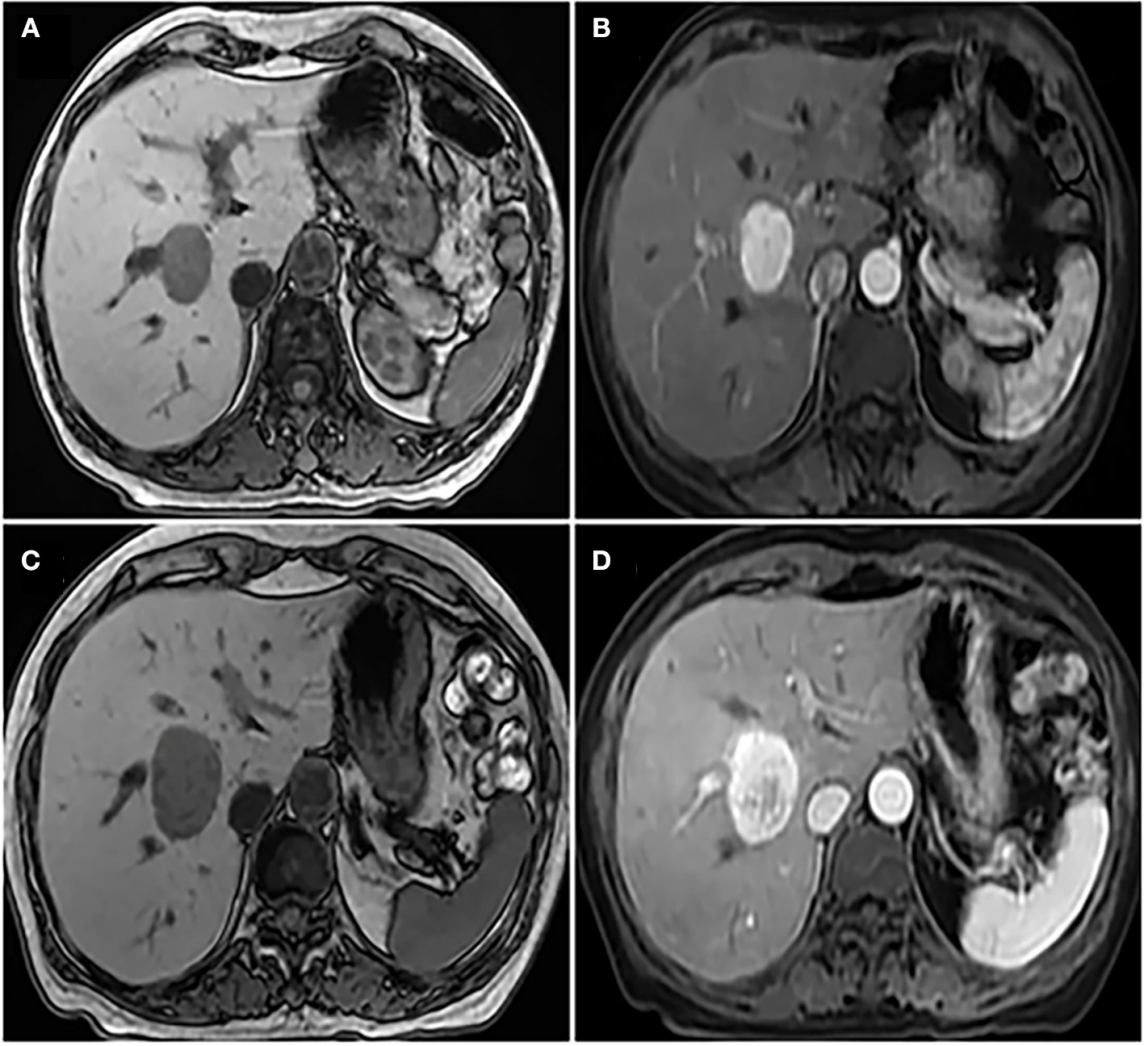
**(A)** Non-enhanced abdominal MRI showed that there was an occupying mass in the right lobe of liver, the boundary of tumor was unclear; **(B)** enhanced abdominal MRI indicated mild heterogeneous enhancement of solid components around the tumor and the size of the tumor was ~2.7 × 2.8 × 3 cm; **(C)** a second non-enhanced abdominal MRI showed that the mass in the right lobe of liver grew larger, and the size was ~3 × 3.5 × 3.5 cm; **(D)** a second enhanced abdominal MRI showed the low density of the cyst was seen in the tumor, which was considered to be accompanied by hemorrhage.

**Figure 2:**
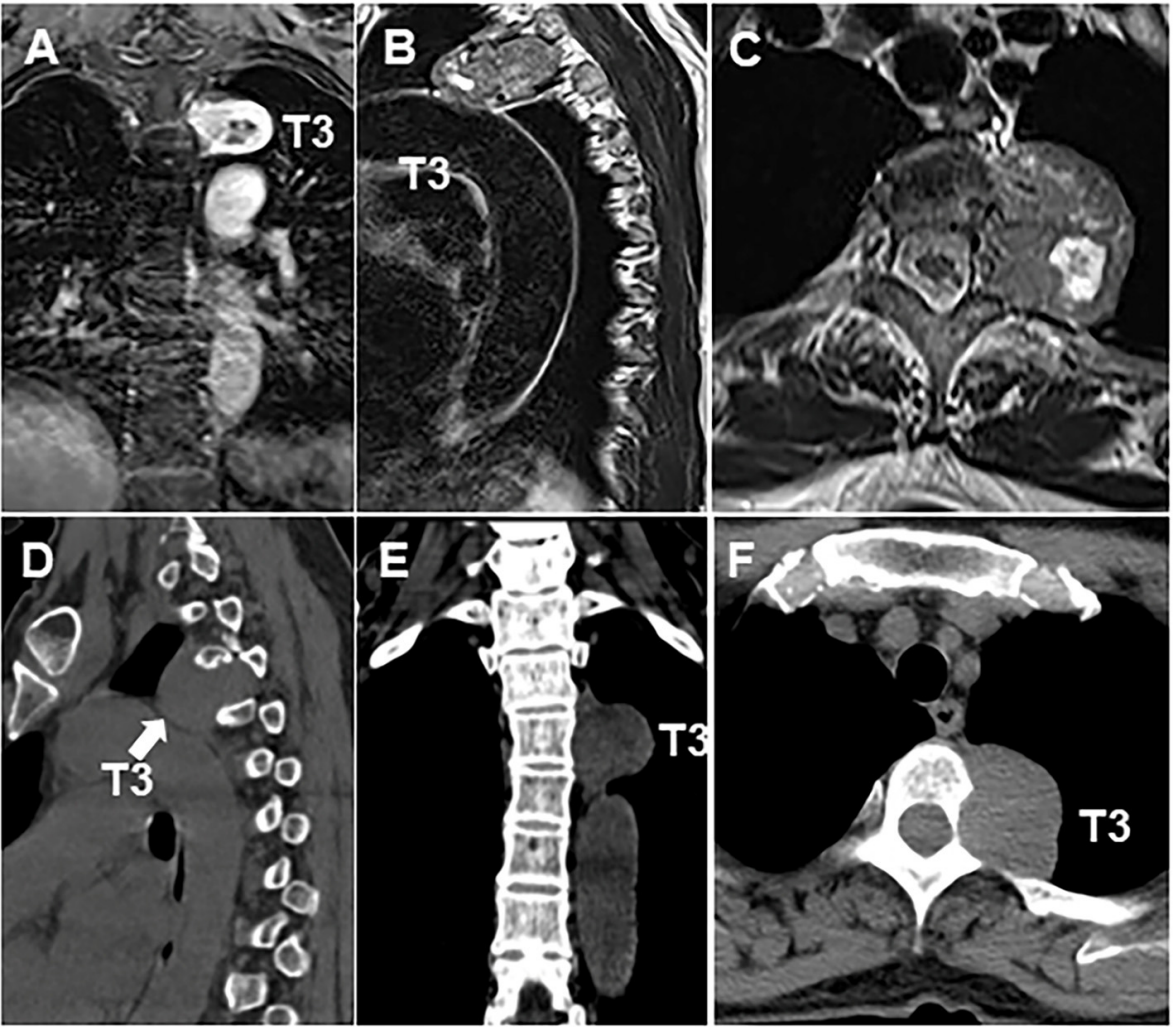
Thoracic MRI and CT scan showed the size of the lesion to the left of the T3 **(A–F)** vertebral body was ~3.7 × 5.3 cm. Arrow indicates the location of tumor.

**Figure 3:**
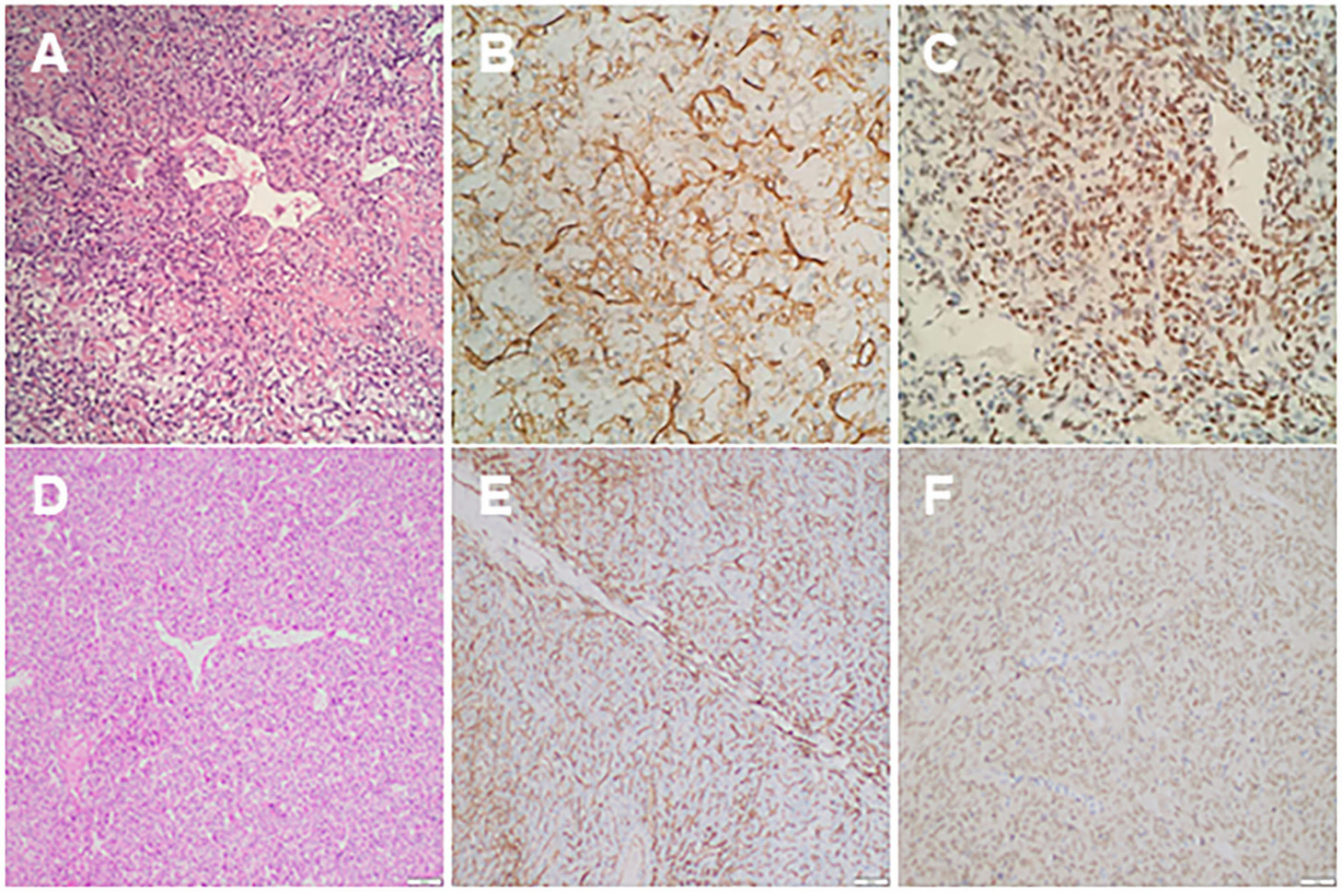
**(A)** Haematoxylin and eosin (HE) stain of thoracic mass showed compact cells with some prominent dilated vessels; **(B)** immunohistochemical study demonstrated the expression of CD34; **(C)** the immunohistochemical study showing positive nuclear reactivity of STAT6; **(D)** HE stains of the liver mass showed compact tumor cells with some prominent dilated vessels; **(E)** immunohistochemical study demonstrated the expression of CD34; **(F)** immunohistochemical study showed positive nuclear reactivity of STAT6.

**Figure 4:**
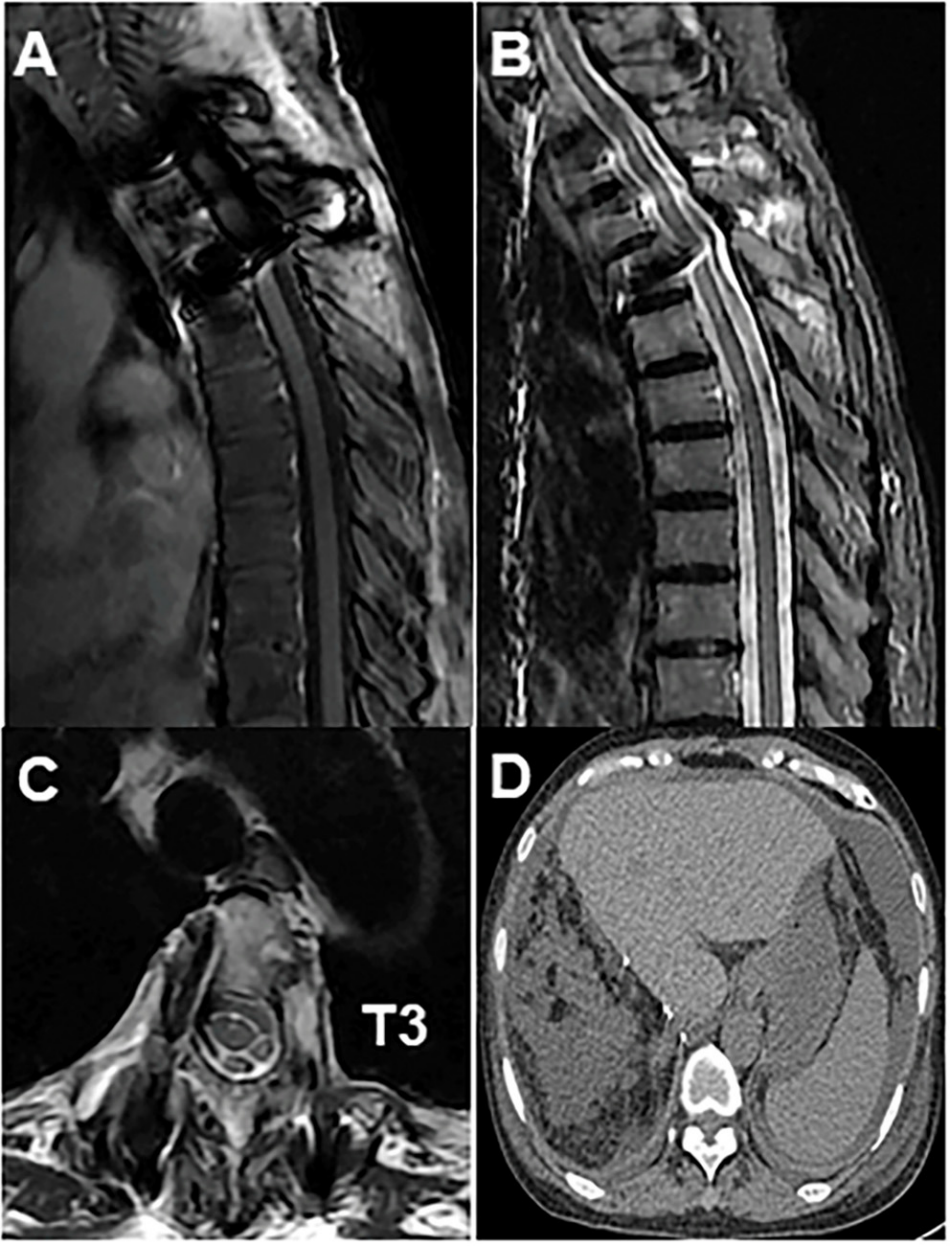
**(A)** Post-operative radiograph showed fixation of the thoracic spine from T2 to T4; **(B)** T2-weighted sagittal MRI showed no disease recurrence at T3; **(C)** axial MRI showed no disease recurrence at T3; **(D)** abdominal MRI showed no disease recurrence at the liver.

The patient is a 60-year-old woman who presented to our hospital with new-onset upper back pain. The patient complained of upper back pain for 6 months accompanied by left intercostal neuralgia, which was refractory to analgesic treatment. The patient underwent gross total resection of a meningeal tumor 10 years ago. The pathology result was a hemangiopericytoma with a high proliferation index that was classified as WHO grade 2. The patient received twenty-seven cycles of adjuvant radiotherapy after surgical resection. Then, the patient had an excellent post-operative course, and yearly follow-up computed tomography (CT) scans confirmed a complete resection and no recurrence of the tumor until March 2017. Ten years after resection of the meningeal tumor, her upper back pain started. First of all, the patient admitted to another hospital, the positron emission tomography/computed-tomography (PET-CT) showed the lesion at the left T3 vertebral body and in the right lobe of the liver without other sites metastasis. The diagnosis of metastatic SFT was suggested by immunohistochemistry of the T3 mass after CT-guided percutaneous thoracic puncture biopsy was performed. The immunohistochemical study demonstrated CD34(+) and STAT6(++). Then, abdominal and thoracic magnetic resonance imaging (MRI) were performed. The abdominal MRI revealed an enhanced lesion in the right lobe of the liver, with a size of ~2.7 × 2.8 × 3 cm (Figures 1A,B). The patient complained of no discomfort in the abdomen and did not present with jaundice. The thoracic MRI showed an expansive intraosseous mass at the left T3 vertebral body. The bony mass expanded along the left of the T3 vertebral body, with retropulsion toward the spinal canal and compression of the spinal cord at this level (Figures 2A–C). The thoracic CT scans showed that the size of the lesion to the left of the T3 vertebral body was ~3.7 × 5.3 cm (Figures 2D–F). The Tomita score was 4, and the Tokuhashi score was 9–11, indicating long-term control requiring wide or marginal excision. On the basis of these findings, the surgical plan included marginal excision of the T3 tumor with T2-4 pedicular screw fixation (Figure 4A). The surgery was performed smoothly without complications or significant blood loss. The preoperative symptom of back pain was relieved, and a radiograph of the thoracic spine showed proper fixation. Then, the diagnosis of SFT was suggested by immunohistochemistry performed on the spinal mass. The immunohistochemical study demonstrated CD34(+) and STAT6(+) (Figures 3A–C). The patient had an excellent post-operative recovery. After 3 months, a second abdominal MRI showed that the mass in the right lobe of the liver grew larger, and the size was ~3 × 3.5 × 3.5 cm (Figures 1C,D). A right hemi-hepatectomy was performed to remove the liver lesion by gross total resection. The diagnosis of SFT was confirmed with the expression of CD34 and nuclear expression of STAT6 by immunohistochemistry performed on the liver mass (Figures 3D–F). As the prognosis of malignant and metastatic SFTs was poor, we suggested the patient underwent the adjuvant radiotherapy after complete resection of thoracic and liver metastasis SFT. But the patient refused adjuvant radiotherapy and carried out post-operative follow-up on time. Two-year follow-up thoracic MRI and abdominal CT revealed no disease recurrence at the T3 (Figures 4B,C) and the liver (Figure 4D).

## Discussion

SFTs are rare neoplasms that originate from the pleura. However, SFTs have been found to occur in any site, including the spine. Spinal SFTs are always classified as intramedullary (58%), intradural extramedullary (24%), or extradural (18%) (9, 10). More importantly, as far as we know, no previous reports have described a malignant and metastatic SFT occurred in the thoracic vertebrae. SFTs may occur in individuals of all ages, ranging from 10 to 85 years. Although most of SFTs have benign features, 10–20% of SFTs are malignant and prone to metastasis. Many SFT patients probably need to undergo repeated surgery, radiotherapy and chemotherapy because of high recurrence rate and the risk of distant metastasis.

Histopathologically, typical benign SFTs contain clear tumor cell boundaries and dilated branched blood vessels. Moreover, typical benign SFTs are always present with a fusiform pattern, scattered among the collagen fiber bundles in different forms. In addition, 80% of extra thoracic SFTs always present with atypical histological features, including increased cellularity, a large number of nuclear pleomorphisms, focal necrosis, and four or more mitoses per ten high-power fields (HPFs). Most SFTs usually exhibit positive staining for mesenchymal and vascular endothelium-related antigens, such as CD34, CD99, Bcl-2, and vimentin, whereas these tumors exhibit negative staining for SMA, EMA, and GFAP. Because these biomarkers are non-specific for the diagnosis of SFTs, STAT6 was recently found to be a typical tumor marker for the diagnosis of SFTs (11, 12). Therefore, the combination of STAT6 with CD34 plays a crucial role in diagnosing SFTs. More importantly, the presence of malignant cellular features in SFTs significantly increases the possibility of local recurrences or distant metastases.

Furthermore, as reported by Melone et al., high-grade SFTs recur 90% of patients, whereas the rate of recurrence for low-grade SFTs was merely 27% (13). Statistically, the mean time of distant metastasis is ~7.5 years, which indicates that long-term follow-up is required for the treatment of SFTs. Moreover, once distant metastasis occurs, the prognosis of SFTs worsens. In our case, although the primary meningeal SFT was completely resected and did not recur based on post-operative brain CT, the patient experienced distant metastasis after 10 years, including to T3 and the liver. To the best of our knowledge, previous reports of SFT metastasis to both of the spine and liver are relatively rare, with equal distribution among the cervical, thoracic, and lumbar spinal regions. Additionally, the vertebral body is the most likely metastasis site because of its abundant vascularization and bone marrow.

In terms of the management of metastatic SFTs, there is still no consensus because of the lack of sufficient research with long-term follow-up in the literatures. The management of metastatic SFTs typically consists surgery with or without radiotherapy. However, surgical excision still remains the first choice of treatment. Son et al. reported a case of malignant SFT which was resected but a local recurrence developed rapidly (14). Meanwhile, as reported by van Houdt et al., the factor most significantly correlated with local recurrence is the positive resection margin (15). Therefore, patients with a positive excision margin have a statistically significant higher risk of local recurrence. Some authors claim that maximal safe surgical excision remains the optimal treatment for SFTs. As reported, gross total resection is better than subtotal resection and leads to a longer disease-free survival (16).while some authors claim that en-bloc resection is the best treatment for SFTs (17, 18). In our case, the presented patient underwent gross total resection at the T3 vertebrate and liver sites. Post-operative radiotherapy has been shown to prolong the median progression-free survival from 44 to 58 months and overall survival from 80 to 93 months. Although the role of post-operative radiotherapy is not well-established, it can be used in patients with residual disease, high-grade tumors, and metastatic disease. Nonetheless, post-operative radiotherapy currently remains an adjuvant method rather than a substitute for gross total resection because gross total resection alone has a superior median overall survival compared to subtotal resection. As the prognosis of malignant and metastatic SFTs is poor, post-operative follow-up is highly recommended. More importantly, long-term clinical or radiographic follow-up remains mandatory to monitor a late recurrence or metastasis which can occur after more than 10 years (19, 20).

## Conclusion

The literature lacks randomized controlled trials and large studies that define the natural history of malignant SFTs and recommendations of a precise management plan for the disease. Further studies are needed to understand the long-term outcome and clinical features for the diagnosis of the rare clinical situation. However, the best choice for treatment is gross total resection, which seems to provide the optimal treatment to achieve long-term disease-free survival.

## Data Availability Statement

The original contributions presented in the study are included in the article/supplementary materials, further inquiries can be directed to the corresponding author/s.

## Ethics Statement

Written informed consent was obtained from the individual(s) for the publication of any potentially identifiable images or data included in this article.

## Author Contributions

MM and LZ contributed equally to the writing of the manuscript and designed the figures. CH and XY collected clinical data and followed the patient during the clinical course. SH revised the article. HY and QZ interpreted the radiological images. DS reviewed and approved the final version of this article. All authors contributed to the article and approved the submitted version.

## Conflict of Interest

The authors declare that the research was conducted in the absence of any commercial or financial relationships that could be construed as a potential conflict of interest.
